# Cardiovascular Functional Changes in Chronic Kidney Disease: Integrative Physiology, Pathophysiology and Applications of Cardiopulmonary Exercise Testing

**DOI:** 10.3389/fphys.2020.572355

**Published:** 2020-09-15

**Authors:** Kenneth Lim, Gordon McGregor, Andrew R. Coggan, Gregory D. Lewis, Sharon M. Moe

**Affiliations:** ^1^Division of Nephrology, Indiana University School of Medicine, Indianapolis, IN, United States; ^2^Coventry University Hospital, Coventry and Warwickshire NHS Trust, Coventry, United Kingdom; ^3^Warwick Clinical Trials Unit, Warwick Medical School, University of Warwick, Coventry, United Kingdom; ^4^Department of Kinesiology, Indiana University – Purdue University, Indianapolis, IN, United States; ^5^Division of Cardiology, The Massachusetts General Hospital and Harvard Medical School, Boston, MA, United States

**Keywords:** cardiopulmonary exercise testing (CPET), cardiovascular functional capacity, VO_2_Peak, chronic kidney disease (CKD), end-stage kidney disease (ESKD), dialysis

## Abstract

The development of cardiovascular disease during renal impairment involves striking multi-tiered, multi-dimensional complex alterations encompassing the entire oxygen transport system. Complex interactions between target organ systems involving alterations of the heart, vascular, musculoskeletal and respiratory systems occur in Chronic Kidney Disease (CKD) and collectively contribute to impairment of cardiovascular function. These systemic changes have challenged our diagnostic and therapeutic efforts, particularly given that imaging cardiac structure at rest, rather than ascertainment under the stress of exercise, may not accurately reflect the risk of premature death in CKD. The multi-systemic nature of cardiovascular disease in CKD patients provides strong rationale for an integrated approach to the assessment of cardiovascular alterations in this population. State-of-the-art cardiopulmonary exercise testing (CPET) is a powerful, dynamic technology that enables the global assessment of cardiovascular functional alterations and reflects the integrative exercise response and complex machinery that form the oxygen transport system. CPET provides a wealth of data from a single assessment with mechanistic, physiological and prognostic utility. It is an underutilized technology in the care of patients with kidney disease with the potential to help advance the field of cardio-nephrology. This article reviews the integrative physiology and pathophysiology of cardio-renal impairment, critical new insights derived from CPET technology, and contemporary evidence for potential applications of CPET technology in patients with kidney disease.

## Introduction

Cardiovascular disease is a modern-day global epidemic (Kwan and Benjamin, 2015). Over the past century, our world has witnessed a striking epidemiologic transition in the predominant cause of death, from communicable diseases and nutritional deficiencies to non-communicable diseases (Nascimento et al., 2014). At the forefront of non-communicable conditions are diseases of the cardiovascular system. A number of models that include health behaviors, population aging, and increasing rates of urbanization and globalization that increase the burden of cardiovascular risk factors, partially account for this epidemiologic transition in cardiovascular disease (CVD) (Yusuf et al., 2001). Other well-established risk factors for CVD include hyperlipidemia, hypertension, smoking and diabetes. However, in recent years, Chronic Kidney Disease (CKD) has emerged as a risk factor of considerable importance (Sarnak et al., 2003). In fact, CVD is now well-recognized to be the leading cause of death in CKD patients. Individuals with only mild decrements in glomerular filtration rate (GFR) to <60 ml/min/1.73 m^2^ have already a two-fold increased risk of cardiovascular mortality and this risk increases up to 20-fold by the time a patient needs renal replacement therapy, or end-stage kidney disease (ESKD) (Gansevoort et al., 2013). The pattern of overt CVD in CKD patients differs substantially from the general population. Occlusive coronary artery disease (CAD) accounts for only a minority of cardiovascular deaths in advanced CKD, with the majority being attributed to sudden cardiac death (SCD) and congestive heart failure, in contrast to the general population [Wilson et al., 2001; Wayhs et al., 2002; US Renal Data System (USRDS), 2013].

**FIGURE 1:**
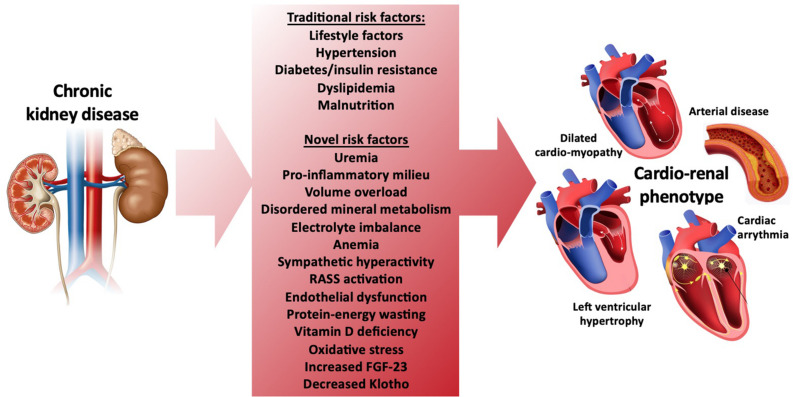
Traditional and novel risk factors for development of cardiovascular disease and the cardio-renal phenotype in CKD.

As members of the scientific and health care community, we are challenged with an ethical and moral obligation to help reduce premature mortality from CVD in our growing CKD population. However, the challenge of helping to improve patient outcomes in this population is compounded by the highly complex processes involved in CVD development as kidney failure ensues. As renal function declines, the development of CVD involves both traditional and non-traditional risk factors such as uremia, pro-inflammatory cytokines, volume overload, mineral disorders, electrolyte disturbances, anemia, sympathetic nerve activation, renin-angiotensin-aldosterone (RAAS) activation and vitamin D deficiency. Additionally, emerging risk factors such as elevated fibroblast growth factor (FGF)-23, low Klotho levels, post-translational protein modifications, and gut derived uremic toxins have now been tightly linked with CVD development in CKD (Rhee and Gerszten, 2012; Kahn et al., 2013; Verbrugge et al., 2015) (Figure 1). Central to these processes are complex interactions between multiple target organ systems that include the renal, musculoskeletal, pulmonary, gastrointestinal, vascular, and cardiovascular systems. Interactions between these organ systems underlie critical homeostatic processes such as endocrine loops that tightly regulate mineral metabolism and other organ cross-talk. Their collective failure originates from kidney dysfunction and contributes to overall cardiovascular impairment (Ting et al., 2015; Lim et al., 2020). These organ system interactions, therefore, provide strong rationale for an integrated physiologic approach to assessing cardiovascular changes in CKD.

The striking multi-tiered, multi-dimensional complexities of CVD development in CKD have challenged our diagnostic and therapeutic efforts. For example, imaging of single organs such as the heart by echocardiography or MRI does not adequately reflect the complex systemic processes that lead to cardiovascular impairment in CKD and is therefore insufficiently sensitive to accurately reflect cardiovascular health in this population. Despite huge scientific, economic and financial investment, many of our cardio-renal outcome trials in nephrology have yielded neutral results and have not demonstrated a treatment benefit (Lim et al., 2018). This may be secondary to multiple reasons such as patient heterogeneity, complexity of cardio-renal pathophysiology, competing risks and importantly, limitations of resting cardiac geometric endpoints for tracking disease improvement or decline in the CKD population. As a result, clinicians are left with potentially equivocal recommendations and patients are left without evidence-based guidance to manage their condition. This state of the union therefore demands alternative or novel approaches to investigative, diagnostic and therapeutic efforts to help combat the burden of cardiovascular disease in CKD.

The emergence of state-of-the-art Cardiopulmonary Exercise Testing (CPET) technology and its application to cardiovascular disease research in nephrology is one such alternative approach gaining significant traction. While only a handful of studies have applied CPET technology to study patients with CKD, these studies have already revealed significant mechanistic and physiological insights, and have provided evidence to support the potential use of CPET-derived indices in a variety of applications in nephrology. This article considers integrative physiological and pathophysiological insights into cardiovascular impairment in CKD derived from CPET technology, and appraises contemporary evidence for the potential application of CPET to help advance the field of cardio-nephrology.

## Integrative Physiology and the Fick Principle

### The Fick Principle and the Fick Equation

As CKD progresses, the integrated metabolic machinery required for the cardiovascular system to function and enable optimal exercise performance is impaired. At the level of the heart and vasculature, kidney failure leads to a uremic phenotype that recapitulates many features of cardiovascular aging, including myocyte hypertrophy, reduced myocardial capillarization and non-vascularized myocardial interstitial fibrosis and calcification as well as vascular calcification, arteriosclerosis and arterial stiffening of systemic vasculature (Aoki et al., 2005; Edwards et al., 2014). The molecular, ultrastructural and geometric changes of the heart and vasculature collectively lead to reduced cardiac efficiency and hence increased myocardial energy expenditure and oxygen consumption. Unfortunately, cardiac remodeling occurs early in the course of CKD leading to left ventricular (LV) diastolic then systolic dysfunction and sudden cardiac death (Edwards et al., 2014; Rutherford and Mark, 2017).

**FIGURE 2:**
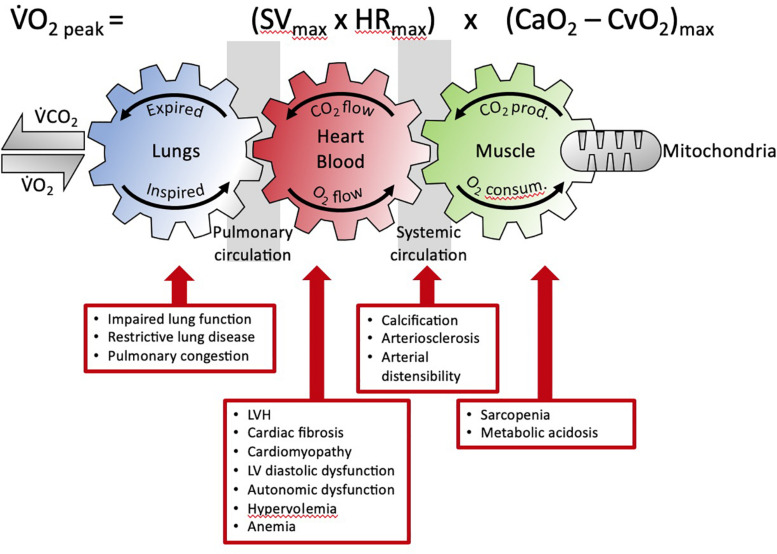
The Fick equation and the coupling of external and cellular respiration. The three interlinked gears represent the functional interdependence between the lungs, circulation and muscle. This facilitates O_2_ transport from the lungs to the mitochondria and, in reverse, CO_2_ from the muscle to the lungs (adapted from Wasserman, 1997). The detrimental multisystemic effects of kidney disease on this integrated physiological process are indicated. V̇O_2_, oxygen uptake; V̇CO_2_, carbon dioxide output; prod, production; consum, consumption; SV, stroke volume; HR, heart rate; CaO_2_, arterial O_2_ content; CaO_2_, venous O_2_ content; LVH, left ventricular hypertrophy.

Critically, CKD results in the failure of multiple organ systems beyond alterations to the heart and vasculature (Figure 2) (Walsh, 1997; Wasserman, 1997; Moorthi and Avin, 2017; Lim et al., 2018): The musculoskeletal system is subject to impairment as CKD progresses resulting in biomechanical failure. This involves muscle wasting (sarcopenia) and alterations in bone mineral metabolism occur leading to widespread consequences, including increased risk of bone mineral disorders (BMD), falls and frailty, hospitalizations, and poorer quality of life (Moorthi and Avin, 2017). Significant interactions between the kidneys and the lungs are also well-known. For example, pulmonary-renal syndromes due to small-vessel vasculitis can cause significant renal impairment, including rapidly progressive glomerulonephritis (RPGN) and pulmonary hemorrhage. Diabetes can cause diabetic nephropathy as well as impaired lung function, involving decreased lung diffusion capacity and increased risk for pulmonary hypertension (Sorino et al., 2019). Additionally, the development of restrictive lung disease has been associated with progressive CKD (Mukai et al., 2018). Underlying these systemic target organ changes are widespread ultrastructural and molecular alterations that contribute to subclinical disease early in the course of CKD and may not be readily detected with conventional resting imaging studies (Ting et al., 2015; Kim et al., 2018; McGregor et al., 2018).

These diverse systemic alterations contribute to the impairment of the cardiovascular system to fulfill its primary function, that is, to be an effective oxygen transport system (Dunn et al., 2016). The role of the many individual components of the cardiovascular system to function effectively in oxygen transport is defined by the Fick Principle. This principle was first described by the German physiologist, Adolf Fick in 1870 and for over 150 years has remained one of the most solid fundamental principles of human cardiovascular physiology (Albouaini et al., 2007). The Fick principle (mathematically expressed by the Fick equation) states that oxygen uptake (VO_2_) equals cardiac output multiplied by the arterial minus venous oxygen content, as illustrated in Figure 2.

The resting oxygen uptake of a healthy individual in a sitting position approximately equals 3.5 ml/min^–1^/kg^–1^ or one metabolic equivalent (MET). However, it has now become clear that resting cardiac and pulmonary function testing cannot reliably predict exercise performance and functional capacity, and that overall health status is more strongly associated with exercise tolerance than with resting measurements (Albouaini et al., 2007). This understanding, together with the principle that the fundamental role of the cardiovascular system is to function as an effective oxygen transport system, provides the rationale for the assessment of oxygen uptake (VO_2_) at maximal or peak exercise (VO_2_Peak) as a robust, objective and reproducible index of cardiovascular functional capacity. While the definition of VO_2_Max and VO_2_Peak are different and will be discussed in further detail below, for the purposes of this article we will use the term VO_2_Peak (unless where VO_2_Max has been explicitly referred too in an original article). VO_2_Peak reflects the maximal ability of an individual to take in, transport and use oxygen and defines the individual’s functional aerobic capacity (Mezzani, 2017).

### Applying the Fick Principle to Patients With CKD

Understanding the Fick equation is of critical importance in order to appreciate how progressive CKD alters cardiovascular function in this population, and thus the utility of functional testing. Lessons learned from the Fick equation, as applied to the complex systemic changes that occur with declining kidney function, demand that we look beyond cardiac or vascular changes alone, toward an integrative viewpoint when assessing overall cardiovascular health status in CKD patients rather than static single-organ measures. For example, left ventricular hypertrophy (LVH) is highly prevalent in the dialysis population and is associated with high rates of all-cause and cardiovascular mortality (Silberberg et al., 1989; Foley et al., 1995; London, 2002). However, not all incident dialysis patients have LVH; in fact, approximately 20% do not. Furthermore, a small proportion of dialysis patients experience some regression, and daily dialysis compared to thrice weekly can prevent worsened LVH (Group et al., 2010). Still, dialysis patients who do not have LVH or exhibit regression continue to have elevated risk of cardiovascular mortality (Charytan, 2014). Similarly, kidney transplantation is associated with improved cardiovascular survival (Meier-Kriesche et al., 2004); however, serial cardiac magnetic resonance (CMR) imaging which provides accurate and reproducible assessment of cardiac dimensions, has failed to identify significant regression in LV mass after transplantation (Patel et al., 2008). Taken together, these clinical observations therefore suggest that measures of LV geometry may not accurately reflect the risk of premature cardiovascular death in advanced CKD.

Importantly, an alteration of any of the four variables in the Fick equation that determines VO_2_ peak can occur in CKD. As an example, in advanced CKD patients, a reduction in maximal heart rate or a blunted chronotropic response occurs, leading to reduced cardiac output (Ting et al., 2015). These maladaptive processes coupled with widespread arterial calcification and stiffening that contribute to increased afterload, lead to further reductions in cardiac output (Ferro et al., 2012). Impairment of the musculoskeletal system from sarcopenia with muscle mitochondrial dysfunction and progressive anemia are common complications of advanced CKD, can also have profound effects on maximal arterial minus mixed venous oxygen content (Cao_2_ – Cvo_2_max) (Lim et al., 2020). All pathophysiological states that impair oxygen transport from air to mitochondria, and oxygen use during exercise can contribute to cardiovascular dysfunction and reduced cardiovascular functional capacity. This makes the assessment of VO_2_Peak and other cardiovascular functional variables particularly powerful measures for assessing cardiovascular health. Reduction in VO_2_Peak has been observed not only in several different organ or systemic conditions (such as chronic heart failure and chronic obstructive pulmonary disease), or conditions that affect the musculoskeletal system (such as mitochondrial myopathies and amyotrophic lateral sclerosis) and are too numerous to completely list here, but have also been described in bed-rest and deconditioning (Mezzani, 2017).

## Cardiopulmonary Exercise Testing (CPET)

Cardiopulmonary exercise testing is a powerful, dynamic technology that incorporates ventilatory gas exchange measurements during graded exercise. CPET assesses gas exchange measures of O_2_uptake (VO_2_), carbon dioxide output (VCO_2_) and minute ventilation (V_E_) and some CPET systems, can provide breath-by-breath analysis of these variables. These measures are used to derive various other gas exchange patterns and can provide organ-specific information on the dysregulated responses to exercise. In the general heart failure population, CPET-derived indices have been recognized as robust markers for the assessment of cardiovascular disease compared to conventional resting imaging for several reasons. Firstly, the coupling of morphological cardiac alterations to cardiovascular performance is largely unknown; secondly, resting cardiopulmonary imaging tests cannot reliably predict functional performance; and thirdly, there is increasing appreciation that overall health status correlates better with exercise tolerance (American Thoracic Society and American College of Chest Physicians, 2003). Here, we will provide a brief discussion of major CPET variables and a summary of commonly derived CPET data is provided in Table 1.

**TABLE 1:** Summary of CPET variables and description.

Abbreviation	Parameter	Units	Description
WR	Work rate	Watts (W)	Work per unit time (where work represents the movement resulting from force being exerted against a mass, e.g., cycle ergometry).
RPE	Rating of perceived exertion		Self-reported subjective measure of the sensations of exertion being experienced by the participant.
t	Endurance time	s	Total exercise time (excluding warm-up).
HR	Heat rate	min^–1^	Number of heart beats (cardiac cycles) per minute.
HRR	Heart rate reserve	min^–1^	Difference between the predicted maximal heart rate and actual peak heart rate achieved.
SBP	Systolic blood pressure	mmHg	
DBP	Diastolic blood pressure	mmHg	
RPP	Rate pressure product	–	Product of HR and SBP. Indirect measure of myocardial work.
FEV_1_	Forced expiratory volume in one second	l	Volume of air expelled from the lungs during first second of forced expiration (measured at rest).
FVC	Forced vital capacity	l	Maximal volume of air expelled from the lungs after maximal inspiration (measured at rest).
BF	Breathing frequency	min^–1^	Number of breaths (ventilatory cycles) per minute.
V_T_	Tidal volume	l	Volume of air inhaled or exhaled in a single breath.
V̇_E_	Minute ventilation	l.min^–1^	Volume of air inhaled or exhaled per minute.
MVV	Maximum voluntary ventilation	l.min^–1^	Maximal ventilatory ability measured by repeated maximal inspiration and expiration over a given time period, e.g., 10 s).
BR	Breathing reserve	%	Difference between resting MVV and maximal V_E_ during exercise. Represents remaining capacity to increase ventilation at maximal exercise.
V̇O_2_	Oxygen uptake	ml.min^–1^ ml.kg^–1.^min^–1^	Volume of O_2_ uptake per minute measured in expired air.
V̇O_2_ max	Oxygen uptake at maximal exercise	ml.min^–1^ ml.kg^–1.^min^–1^	Highest O_2_ uptake achievable during incremental exercise.
V̇O_2_ peak	Oxygen uptake at peak exercise	ml.min^–1^ ml.kg^–1.^min^–1^	Highest O_2_ uptake achievable during incremental exercise in the context of any physiological limitation (often used synonymously with V̇O_2 max_).
V̇O_2_ % pred	Oxygen uptake as a percentage of predicted	%	Highest O_2_ uptake achieved during incremental exercise relative to predicted value (values of 80–120% predicted are considered normal).
V̇CO_2_	Carbon dioxide output	ml.min^–1^	Volume of CO_2_ exhaled per minute measured in expired air.
RER	Respiratory exchange ratio	–	Ratio of CO_2_ output to O_2_ uptake measured in expired gas.
VAT	Ventilatory anaerobic threshold	ml.kg^–1.^min^–1^	O_2_ uptake at the ventilatory anaerobic threshold.
VAT % pred. V̇O_2 peak_	Ventilatory anaerobic threshold as a percentage of predicted V̇O_2_ peak	%	Oxygen uptake at the ventilatory anaerobic threshold relative to predicted V̇O_2 peak_. Values below 40% are generally considered indicative of pathology.
P_ET_O_2_	End tidal partial pressure of O_2_	mmHg	Partial pressure (tension) of O_2_ in exhaled gas at the end of expiration
P_ET_CO_2_	End tidal partial pressure of CO_2_	mmHg	Partial pressure (tension) of CO_2_ in exhaled gas at the end of expiration
O_2_ pulse	Oxygen pulse	ml	Amount of O_2_ extracted by tissue per heart beat (i.e., stroke volume) Measure of overall cardiovascular efficiency.
OUES	Oxygen uptake efficiency slope	–	Regression-derived variable representing the relationship between log-transformed V_E_ and V̇O_2_. Measure of overall cardio-pulmonary function.
ΔV̇O_2_/ΔWR slope	Oxygen uptake/work slope	–	Increase in O_2_ uptake in relation to a simultaneous increase in work rate. Lower values indicate inability to augment V̇O_2_ in response to increase in WR.
V̇E/V̇O_2_	Ventilatory equivalent for O_2_	–	Volume of O_2_ uptake per unit of ventilation.
V̇E/V̇CO_2_	Ventilatory equivalent for CO_2_	–	Volume of CO_2_ output per unit of ventilation.
V̇E/V̇CO_2_ slope	Slope of the ventilatory response	–	The slope of the response of the ventilatory equivalent for CO_2_. Measure of ventilatory efficiency (ventilation/perfusion matching).
Sao2	Oxyhemoglobin saturation	%	Oxyhemoglobin saturation measured by pulse oximetery

### Oxygen Uptake and Peak Oxygen Uptake

**FIGURE 3:**
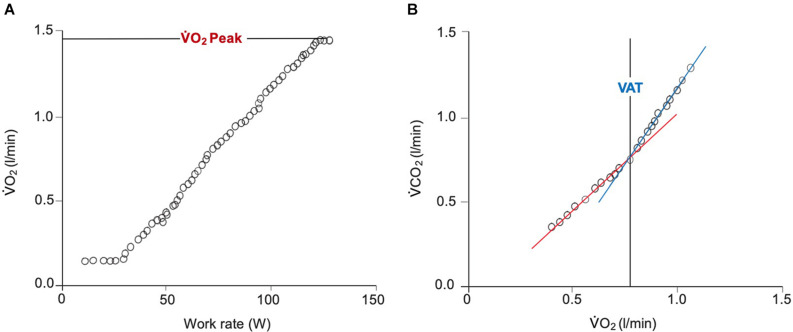
Breath-by-breath gas exchange measurements during a ramp protocol CPET. **(A)** Demonstrates the linear increase in V̇O_2_ in response to a linear increase in work rate. Achievement of V̇O_2_Peak is confirmed by the plateau in V̇O_2_ beginning at approximately 125 W. **(B)** Displays the derivation of the VAT using the ‘V-slope’ method. The point at which the lower (blue) and upper (red) slopes intersect indicates the VAT. V̇O_2_, oxygen uptake; V̇CO_2_, carbon dioxide output; VAT, ventilatory anaerobic threshold.

Maximum oxygen uptake (VO_2_Max) is defined as the highest rate of oxygen uptake during intense, maximal exercise whereby no further increases in work rate can cause additional rises in VO_2_ (i.e., plateau) (Bassett and Howley, 2000). Peak VO_2_ (VO_2_Peak) is directly reflective of VO_2_Max and is defined as the highest value of VO_2_ obtained upon an incremental or other high-intensity exercise test that brings the individual to the limit of tolerance (Whipp and Ward, 1990). VO_2_Peak considers the integrative exercise response involving the degree of ventricular function (pumping capacity), oxygen transport in blood (O_2_ carrying capacity), pulmonary and vascular function (O_2_ delivery), skeletal muscle metabolic capacity (O_2_ utilization), and as well as ultrastructural and molecular changes of organ systems involved, and their interactions (Malhotra et al., 2016). During incremental exercise testing, it is defined as the highest volume of VO_2_ averaged over a period of time 20- to 30- s period achieved at presumed maximal effort (Figure 3). The period of which VO_2_Peak is measured isn’t standardized, but generally range between a 20- to 30- s period to a minute depending on investigator preference. VO_2_Peak is quantified in liters or milliliters of oxygen per minute or in milliliters per kilogram of body weight per minute. It is a parameter that describes the maximal amount of energy obtainable by aerobic metabolism per unit time (aerobic power) at peak or incremental exercise (Mezzani, 2017). It is therefore powerfully reflective of cardiovascular functional capacity by taking into consideration maximal exercise tolerance.

On average, VO_2_Peak declines by 10% per decade after the age of 30 and this has been attributed to decreasing maximal heart rate, stroke volume, blood flow to skeletal muscle and skeletal muscle aerobic potential (Betik and Hepple, 2008). Due to higher hemoglobin levels, greater muscle mass and stroke volume, VO_2_Peak is approximately 10–20% greater in men than in women of comparable age (Astrand, 1960). Because VO_2_Peak is influenced by age, gender, and muscle mass, it is therefore appropriate to interpret VO_2_Peak normalized to age, gender and weight-based normative values (Grassi et al., 2009).

### Submaximal Oxygen Uptake Measurements

Although VO_2_Peak has been the most commonly used variable for assessing cardiovascular functional capacity, gas exchange indices obtainable during submaximal exercise have emerged and may rival or even exceed the prognostic utility of VO_2_Peak in various settings (Malhotra et al., 2016). Incremental exercise can be divided into two phases, firstly an initial phase that lasts until 50–60% of VO_2_Peak during which expired ventilation (VE) increasing linearly with VO_2_ and VCO_2_. This is followed by a second phase during which VE increases disproportionately relative to first VO_2_ and then VCO_2_ (Albouaini et al., 2007). This transition point was initially termed the anaerobic threshold (AT), because in healthy subjects it tends to coincide with the exercise intensity at which the rates of glycolysis and especially glycogenolysis accelerate rapidly, leading to accumulation of pyruvate and hence lactate in muscle and blood (i.e., the lactate threshold). It has long been known, however, the muscle is only truly anaerobic at intensities > 100% of VO_2_max. Furthermore, numerous studies have demonstrated that it is possible to dissociate changes in VE from changes in lactate (Poole and Gaesser, 1985; McLellan and Gass, 1989). For example, the muscles of patients with McArdle’s disease lack the ability to produce lactate due to the absence of glycogen phosphorylase activity. Such individuals therefore do not exhibit a lactate threshold, but their VE increases non-linearly with exercise intensity similar to that of a normal person (Hagberg et al., 1990). Similarly, high or low muscle glycogen levels, high or low pedaling rates, different types of exercise training, etc., have all been shown to dissociate changes in VE from changes in lactate (Hughes et al., 1982). The “breakpoint” in VE with increasing exercise intensity is therefore more appropriately described as simply a ventilatory threshold (VT), and can be estimated non-invasively using various quantitative methods, such as the V-slope method. In the V-slope method, VT is defined as the VO_2_ at which the rate of increase in VCO_2_ relative to VO_2_ increases in the absence of hyperventilation (Beaver et al., 1986). Although estimation of VT can be replaced by direct blood sampling to determine the lactate threshold (LT), this is rarely performed in a clinical setting.

Submaximal CPET indices such as VT are attractive due to the relative ease with which they are ascertained during low-level exercise, their independence from volitional effort, and their relevance to an individual’s ability to perform activities of daily living. Ascertainment of submaximal VO_2_ parameters becomes particularly relevant in patients with heart failure who fail to fulfill the criteria for maximum volitional effort, and this is discussed in more detail below. For example, the ability to exercise beyond the VT can help distinguish impaired cardiovascular functional capacity from non-cardiac (pulmonary or musculoskeletal) causes of exercise limitation (Albouaini et al., 2007). However, this is not universally true as patients with mitral stenosis for example, often stop exercising before they reach VT, while patients with chronic obstructive pulmonary disease (COPD) commonly pass the VT.

### Respiratory Exchange Ratio

The respiratory exchange ratio or RER is the ratio between VCO_2_ and VO_2_. During steady-state exercise below VT, RER provides an indication of substrate selection (i.e., fat vs. carbohydrate). Above VT, however, progressive increases in VE result in the liberation of “excess” CO_2_ from bicarbonate stores. This helps to buffer the protons being produced as a result of high rates of glycolysis/glycogenolysis, but invalidates the use of RER as a measure of substrate oxidation. Nonetheless, RER still provides an objective descriptor of subject motivation. An RER greater than approximately 1.0 implies that an individual has attained a maximal volitional effort, whereas an RER less than this value suggests that they have not. Measurement of RER during CPET testing and is therefore of crucial significance to assist in the attainment of reliable and clinically meaningful VO_2_Peak values (Mezzani, 2017).

### Oxygen Pulse

Oxygen pulse is the ratio between VO_2_ and heart rate, and reflects the amount of oxygen consumed per heartbeat. It is a measure for stroke volume and peripheral oxygen extraction during exercise, and can be calculated as stroke volume multiplied by C(a-v)O_2_ (Mezzani, 2017). Flattening or downward displacement of oxygen pulse kinetics during incremental exercise has been shown to be reflective of peripheral vascular perfusion or extraction or central cardiogenic performance limitations (Mezzani, 2017). Under certain conditions, the morphological analysis of its curve can aid in the diagnosis of ventricular dysfunction and exercise-induced myocardial ischemia (Herdy et al., 2016).

## Cardiovascular Functional Changes in CKD

### End-Stage Kidney Disease (ESKD)

In a matched cohort study, Ting et al. (2015) examined cardiovascular functional capacity changes in a study that recruited 80 dialysis patients and 80 hypertensive controls. The authors reported that dialysis patients had a reduction in VO_2_Peak (18 ± 4.1 ml/min^–1^/kg^–1^) compared to controls (24.5 ± 7.1 ml/min^–1^/kg^–1^, *p* < 0.001) (Ting et al., 2015). The study also found that LV ejection fraction was significantly lower in dialysis patients compared to hypertensive controls, however, this was not predictive of VO_2_Peak. Further analysis revealed that LV filling pressure and pulse wave velocity were independent predictors of reduced VO_2_Peak in dialysis patients. Conversely, LV mass index and LV end-diastolic volume were predictive of VO_2_Peak in the hypertensive control group. These results suggest important mechanistic differences leading to reduced cardiovascular function in advanced CKD as opposed to those in hypertensive cardiovascular disease alone. The finding that LV ejection was not predictive of VO_2_Peak is not surprising, given that the majority of CKD patients with heart failure have diastolic rather than systolic dysfunction (Loutradis et al., 2018). It is possible that increased LV diastolic stiffness may in part contribute to impaired cardiovascular functional capacity as CKD progresses; further studies are required to elucidate this. Although myocardial growth and remodeling may be a dynamic, adaptive process that occurs early in the course of CKD, it is likely that sustained cardiac afterloads due to volume overload in ESKD is a major contributor to progressive cardiac remodeling. Additionally, in the study by Ting et al. (2015) dialysis patients exhibited chronotropic incompetence with a reduced maximal heart rate. Taken together, these results suggest that maladaptive LV changes and blunted chronotropic responses are involved in impairment of cardiovascular function in dialysis patients.

The CAPER (Cardiopulmonary Exercise Testing in Renal Failure and After Kidney Transplantation) study by Lim et al. (2020) was the first study to assess cardiovascular functional changes using CPET before and after kidney transplantation. CAPER was a prospective, non-randomized, single-center, 3-arm controlled cohort study that recruited a total of 253 patients: 81 stage 5 CKD patients who underwent kidney transplantation, 85 non-transplanted waitlisted stage 5 CKD patients, and 87 hypertensive controls. All patients underwent CPET and echocardiography assessment at baseline and were followed longitudinally for up to 1 year. In the non-transplanted CKD stage 5 group, who were waitlisted but did not undergo transplantation after 1 year follow-up, CPET was sensitive enough to detect a decline in cardiovascular functional capacity (as assessed by VO_2_Peak) (18.9 ± 4.7 to 17.7 ± 4.1 mL.min^–1.^kg^–1^, *p* < 0.001). These results become even more revealing considering that the study found that LV mass index did not change (*p* = 0.20). This highlights the limitations of using LV mass or hypertrophy as a surrogate endpoint for tracking disease progression in advanced CKD patients, and that other processes beyond LV geometric indices are likely involved in driving overall cardiovascular functional decline in this population.

CAPER found that LV ejection fraction declined after 1 year (*p* = 0.003). Among the variables that were correlated with VO_2_Peak changes from baseline to 12 months in the non-transplanted dialysis group, were LV mass index, LV ejection fraction, and maximal heart rate as well as hemoglobin level (anemia). It is currently unknown how cardiovascular functional capacity alters with increasing dialysis vintage (length of time on dialysis) beyond 1 year and this data will be critical to help inform cardiovascular risk. Additionally, it is unknown how processes such as myocardial remodeling through fibrosis, myocardial arteriole calcification, capillary rarefaction, myocardial stunning and complex circulating factors such as FGF23, Klotho and metabolites modulate VO_2_Peak decline in dialysis. We postulate that these factors may contribute to reduced cardiac compliance and ventricular contractility, loss of arterial elasticity, as well as end-organ damage of the lungs and skeletal muscle system. Further studies are needed to evaluate this.

Data from the CAPER study highlight the complex organ system interactions that are involved in regulating cardiovascular function in advanced CKD patients and provide evidence of the contribution of multiple target organs that collectively regulate the oxygen transport system (Figure 2). Given that CKD severity is associated with restrictive lung disease (Mukai et al., 2018) and that the lungs are an integral part of the oxygen transport system, it is worrying that few studies have comprehensively assessed pulmonary function changes with advancing CKD, and no studies have examined its relative contribution to impaired cardiovascular functional capacity in CKD. Studies that couple CPET with advanced imaging techniques such as Magnetic Resonance Imaging will help to better elucidate background lung, cardiac and arterial changes that occur and their contribution to impaired cardiovascular functional capacity in this population.

Additionally, given that sarcopenia is a feature of advanced CKD, we are in need of further studies to evaluate the relationship between direct measures of skeletal muscle contractile function and segmental lean muscle mass with changes in cardiovascular functional capacity. Of note, exercise intolerance is an important comorbidity in patients with advanced CKD and is associated with arterial stiffening and endothelial dysfunction (Downey et al., 2017). Emerging data has suggested that reduced cardiovascular functional capacity may be improved with exercise training. In a pilot randomized clinical trial, McGregor et al. (2018) demonstrated parallel improvements in isometric quadriceps strength and exercise cardiovascular functional capacity without significant changes in resting echocardiographic cardiac morphologies, following 10-weeks of exercise training among dialysis patients in a randomized, assessor-blinded, controlled study.

CPET has raised many other questions, for example, how cardiovascular functional capacity changes during short and long interdialytic periods, and how the effects of conventional thrice weekly hemodialysis versus intensive hemodialysis and peritoneal dialysis may alter cardiovascular functional capacity. Further studies are needed.

### Kidney Transplant Recipients

In the CAPER study, Lim et al. (2020) reported significantly improved cardiovascular functional capacity as assessed by VO_2_Peak in the kidney transplant group 1 year after transplantation (20.7 ± 5.8 to 22.5 ± 6.3 mL.min^–1.^kg^–1^, *p* < 0.001). However, cardiovascular functional capacity was not restored to the level observed in the control group without CKD, and this was consistent with the incomplete normalization of kidney function with transplantation (mean eGFR 59.1 ± 18.4 mL.min^–1.^1.73 m^–2,^ to the level of CKD stage 3). These findings become all the more meaningful considering that LVMI did not change after kidney transplantation, despite a significant improvement in VO_2_Peak and when contrasted with the inverse direction of change in non-transplanted dialysis patients. Despite unaltered LVMI, the study reported an improvement in LVEF in the transplanted group at 12 months. These latter findings are consistent with a recent study using volume-independent cardiac magnetic imaging that failed to show significant change in LVMI and LVEF after transplantation (Patel et al., 2008).

Among the variables that were correlated with transplanted-associated VO_2_Peak improvement at 1 year in the CAPER study were cardiac variables (improved LV ejection fraction, maximum heart rate) as well as corrected calcium level (Lim et al., 2020). This latter finding of an association between calcium and change in VO_2_Peak is intriguing given that disordered bone mineral metabolism is a cardinal feature of the failing kidney, and that calcium is centrally involved in regulating cardiac myocyte contractility and relaxation (Hohendanner et al., 2013) and may play a role in regulating myocardial function in uremia. The study did not find a change in plasma corrected calcium levels after kidney transplantation and further studies are needed to explore this. Additionally, improvement in VO_2_Peak at 1 year after transplantation in the CAPER study was also associated with improvements in uremia and fluid overload that were already notable at 2 months after transplantation. These results suggest that reversal of cardiovascular molecular and ultrastructural changes takes at least several months before an improvement in cardiovascular function can be detected in kidney transplant recipients. The pattern of cardiovascular functional changes beyond 1 year after kidney transplantation is unknown and further studies are warranted to evaluate this.

### Chronic Kidney Disease (CKD)

Few studies have assessed cardiovascular functional capacity in pre-dialytic CKD patients. In a study by Nelson et al. (2016) the authors analyzed CPET data from 933 pre-operative patients and found that 93/933 (9.97%) had CKD stage 3 (Nelson et al., 2016). The authors reported that patients with CKD stage 3 had a significantly lower VO_2_Peak (mean difference 6%, 95% CI 1-11%, *p* = 0.02), lower peak heart rate (mean difference 9 bpm, 95% CI 3–14%, *p* = 0.03) and impaired heart rate recovery (mean difference 4 bpm, 95% CI 1–7, *p* < 0.001) compared to patients with a GFR > 60. These findings are suggestive of subclinical cardiovascular disease that can be objectively assessed in pre-dialytic CKD patients. Given that microvascular dysfunction has been shown to be involved in cardiovascular disease in CKD and subclinical disease (Bajaj et al., 2020), further studies are needed to determine whether CPET indices could be used as a surrogate for assessing microvascular dysfunction in CKD. To date, it is unknown how cardiovascular functional capacity alters in the early stages of CKD or its natural history across the spectrum of CKD severity. Additionally, further prospective studies are desperately needed to link changes in VO_2_Peak with cardiovascular outcomes in the CKD population. A summary of observational studies and clinical trials that have utilized CPET-derived endpoints in kidney patients is provided in Table 2.

**TABLE 2:** Summary of clinical studies involving kidney patients that have utilized cardiopulmonary exercise testing (CPET) technology.

References	Year	Population	Summary
**Observational studies**			
Weaver et al.	2008	Pediatric patients: stage 2–4 CKD (*n* = 46), renal transplant recipients (*n* = 33), maintenance HD patients (*n* = 12) and age-matched healthy controls (*n* = 33)	VO_2_max is decreased in children with CKD stages 3 to 4, those on hemodialysis and transplant recipients. Lower VO_2_max can be predicted by the presence of diastolic dysfunction, even if systolic function is normal.
Ting et al.	2013	*N* = 70 living donor kidney recipients.	Reduced AT predicts critical care unit admission in patients undergoing kidney transplantation.
De Souza Faria et al.	2013	*N* = 9 healthy adults, *n* = 29 pre-dialytic CKD stages 3, 4, and 5.	VO_2_Peak as well as submaximal exercise tolerance was impaired in pre-dialytic CKD patients.
Fassbinder et al.	2014	*N* = 54 patients with CKD, 27 stage 1 and 27 stage 2.	No statistically significant difference between stage 1 and 2 patients in VO_2_Peak.
Ting et al.	2014	*N* = 240 patients waitlisted for kidney transplantation, and followed for ≤ 5 years.	Patients with AT < 40% of predicted VO_2_Peak had significantly reduced 5-year cumulative survival rate compared to those with ≥ 40%. Among the patients with AT < 40%, those who underwent kidney transplantation had significantly better survival compared with non-transplanted patients.
Ting et al.	2015	*N* = 80 patients with CKD (61 were dialysis dependent) and 80 patients with essential hypertension.	VO_2_Peak was significantly lower in patients in CKD patients compared to hypertensive controls. Maladaptive LV changes and blunted chronotropic responses were mechanistically involved in reduced cardiovascular functional reserve.
Van Craenenbroeck et al.	2016	*N* = 63 CKD stages 1–5 and *n* = 18 healthy controls.	Impaired VO_2_Peak in mild CKD (stages 1–3A) and correlated with eGFR. Pulse wave velocity was one of the strongest independent determinants of VO_2_Peak.
Nelson et al.	2016	*N* = 993 pre-operative patients, that included *n* = 93 CKD stage 3 patients.	Patients with CKD stage 3 had reduced VO_2_Peak.
Rogan et al.	2017	143 CKD stage 5 or 5d patients and 83 hypertensive controls.	CKD patients had reduced VO_2_Peak, and this was a significant independent predictor of the physical component score (PCS) of the SF-36.
Kirkman et al.	2018	*N* = 31 stage 3–4 CKD and 21 matched healthy controls.	VO_2_Peak, AT, maximum heart rate and 1-min heart rate recovery was reduced in CKD stage 3 patients compared to healthy controls. CKD patients had ventilation perfusion mismatching.
**Clinical trials**			
Mustata et al.	2010	CKD patients, *n* = 10 randomized to 12 months of exercise and *n* = 10 to standard of care.	Long-term exercise training improves VO_2_Peak, augmentation index and health-related quality of life in patients with predialysis CKD.
McGregor et al.	2018	*N* = 46 hemodialysis patients randomized to 10 weeks intra-dialytic cycling, intra-dialytic low-frequency electrical muscle stimulation (LF-EMS) or non-exercise control	Ten weeks of intra-dialytic LF-EMS or cycling improved VO_2_Peak and muscular strength.

*HD, hemodialysis; AT, anaerobic threshold.*

## Potential Applications of CPET Technology in Nephrology

CPET is currently widely used for risk stratification, clinical evaluation and other applications in several medical specialities outside of nephrology. The possibility of adopting global indices such as VO_2_Peak in the field of nephrology has been made more feasible due to technological advances in state-of-the-art CPET and the prognostic value of submaximal indices. Here, we will review several of its existing applications and discuss its potential role in kidney patients.

### Comprehensive Endpoints for Cardio-Renal Trials

Given the limitations of current endpoints such as the LV geometric indices discussed above for assessing cardiovascular alterations in CKD, the potential to use comprehensive endpoints such as VO_2_Peak for cardio-renal outcome trials represents an incredibly attractive alternative. There remains strong experimental and observational evidence that adaptations of cardiovascular structure and function can occur bidirectionally, that is, that adverse cardiovascular remodeling can regress with the potential for recovery in kidney patients (Melchor et al., 2002; Wali et al., 2005). Because significant improvement in cardiovascular outcomes has been reported after kidney transplantation (Pilmore et al., 2010), these data suggest that cardiovascular change in CKD is a modifiable process that can potentially be controlled or halted. Moreover, these data call for sensitive endpoints that can accurately track disease progression or improvement. Considering the complex systemic and widespread ultrastructural and molecular alterations that occur in CKD, a comprehensive or aggregate endpoint such as VO_2_Peak, that considers the complexity of cardiovascular and systemic alterations, may therefore be a particularly appropriate index for assessing cardiovascular improvement or decline in the setting of shifting GFR.

The development of LV hypertrophy, and systolic and diastolic dysfunction are well-recognized predictors of worse cardiovascular outcomes in CKD patients (Charytan, 2014). However, as previously noted above, imaging cardiac structure at rest rather than under the stress of exercise, may be insufficiently sensitive to detect impairment of the cardiovascular system due to renal dysfunction, and may not reliably predict functional performance (Ting et al., 2015). Imaging modalities such as dobutamine stress echocardiography (DSE) have been used in CKD patients. However, DSE has a reduced sensitivity of 80% for detecting inducible ischemia in advanced CKD patients (Parnham et al., 2014). This has been suggested to be secondary to blunted chronotropic responses in CKD; LVH with small intracavitary volume that can obscure detection of wall motion abnormalities at stress, and microvascular CAD that can be difficult to appreciate. Additionally, DSE does not consider other organ system interactions that may affect cardiovascular functional capacity in advancing CKD. Furthermore, there is significant intrinsic value in improving cardiovascular exercise capacity in patients (Malhotra et al., 2016). A low VO_2_Peak defines functional aerobic impairment or exercise intolerance, while an improvement in cardiovascular exercise capacity is sensitively reflected by an increased VO_2_Peak (Albouaini et al., 2007). A summary comparing risk stratification methods, and their advantages and disadvantages in CKD is provided in Table 3 (Hakeem et al., 2014; Parnham et al., 2014).

**TABLE 3:** Cardiovascular risk stratification methods in CKD.

	Advantages	Disadvantages
**Serum biomarker assessment, e.g., Troponin T**	Easy to obtain; can sensitively detect myocardial necrosis; FDA approved in patients with ESKD	Lack of specificity, elevated in more than one-third of patients with ESKD, may require extended evaluation
**Exercise stress EKG**	Widely used methodology for ruling in and ruling out CAD with sensitivity ranging between 71 to 97% and specificity between 64 to 90%.	Requires some functional mobility; does not provide measure of cardiovascular functional capacity; patients with abnormal baseline EKG may limit standard testing
**Resting echocardiography**	Widely available, easy to obtain, low cost; allows assessment of LV geometry and ejection fraction	Inaccuracies with echo interpretation; significant volume shifts occur in dialysis patients; most CKD patients have diastolic failure (thereby limiting usefulness of ejection fraction); LV geometric indices do not sensitivity track disease progression in CKD
**Exercise stress echocardiography**	Allows imaging of extent of regional wall motion responses to stress; has better accuracy for detecting significant coronary stenosis ranging from 80 to 90% compared to exercise EKG	Requires some functional mobility; does not provide measure of cardiovascular functional capacity; technique can be challenging
**Dobutamine stress echocardiography (DSE)**	Well-validated and multiple studies have shown incremental prognostic utility over clinical data for demonstrating resting and stress-induced regional WMA	Increased LV mass or concentric remodeling limits sensitivity for subtle WMA
**Cardiac Magnetic Resonance Imaging (CMR)**	Can detect scar pattern and burden, presence of subendocardial scar by delayed enhancement on CMR has been associated with CAD risk factors, depressed LV ejection fraction and severe CAD on angiography	Little data available on prognostic value of myocardial scar pattern and burden using this technique in CKD population; risk of gadolinium-induced nephrogenic systemic fibrosis
**Myocardial perfusion single-photon emission computed tomography (MPS)**	Well validated prognostic tool in CKD; high prevalence of perfusion defects in ESKD patients	Presence of LVH can compromise sensitivity, due to partial volume effect; can have false negative results in multi-vessel disease due to balanced ischemia
**Coronary artery calcium score (CACS)**	Coronary calcification is highly prevalent in CKD; offers incremental predictive value to clinical risk factors	Does not sensitively track overall cardiovascular disease progression or improvement
Cardiopulmonary Exercise Testing (CPET)*	Comprehensive, takes into account alterations within the entire oxygen transport chain in CKD; sub-maximal derived indices can be obtained with relative ease and does not require maximal volitional effort; can be coupled with imaging or invasive techniques that can provide incremental prognostic data	Selects for patients that have some functional mobility; standardization of CPET testing is not yet uniform across different CPET labs

*All risk stratification methods above except for CPET do not reflect overall functional ability and are reflective of only single-organ changes. CAD, Coronary artery disease; LV, left ventricular; WMA, wall motion abnormality.

From a regulatory standpoint, a significant advantage of CPET-derived endpoints is that they have already been used extensively in clinical trials in the general heart failure population (Malhotra et al., 2016). Importantly, the US Food and Drug Administration (FDA) has evaluated and approved new drugs and devices that have utilized CPET-derived endpoints in clinical trials (Malhotra et al., 2016).

### Cardiovascular Risk Stratification and Prognostic Utility

One of the main manifestations of heart failure is exercise intolerance and this varies with severity of disease. Decreased exercise capacity is associated with higher New York Heart Association (NYHA) functional class, worse symptoms, poor quality of life and decreased patient survival. Exercise capacity is reduced even in mild heart failure and is reflective of an inability of cardiac output to increase adequately with mild exertion (Reddy et al., 1988). VO_2_Peak has been shown to be strongly correlated with maximal cardiac output (Mitchell et al., 1958; Ekblom and Hermansen, 1968). In heart failure, the inability of cardiac output to appropriately increase may result in insufficient perfusion of exercising muscles and premature muscle fatigue (Harrington et al., 1997). Because the NYHA classification of functional impairment in heart failure can be inaccurate due to its subjective nature, objective assessment of cardiovascular functional capacity by CPET offers a significant advantage. Additionally, measures of cardiovascular functional capacity obtained under incremental exercise load reflect overall circulatory health and the ability to respond to physiological and pathological cardio-circulatory stresses (Melchor et al., 2002; Ting et al., 2014). Measures of resting central hemodynamics do not correlate well with functional impairment. For these reasons, CPET has become an integral tool for accurately risk stratifying congestive heart failure patients for timely heart transplantation worldwide (Mancini et al., 1991). The identification of heart failure patients at high risk is crucial to guide their management. In addition to providing robust markers for cardiovascular functional capacity and exercise intolerance, CPET permits assessment of the organ system limiting gas exchange. This technology therefore has significant advantages over 6 min walk tests (6MWT) for evaluating exercise limitations.

Indices of cardiovascular functional capacity have been independently associated with survival in multiple settings. Mancini et al. (1991) in a landmark study involving 114 ambulatory patients with heart failure and reduced ejection fraction in the general population, established VO_2_Peak ≤ 14 mL.min^–1.^kg^–1^ as a criterion for which 1 year survival was significantly lower than that achieved through transplantation. Individuals with a VO_2_Peak > 14 mL.min^–1.^kg^–1^ had a 6% 1 year mortality and this suggests that heart transplantation can be safely deferred in this subgroup of symptomatic heart failure patients. VO_2_Peak potently risk stratifies heart failure patients (with reduced ejection fraction and preserved ejection fraction) into Weber classes A, B, C, and D corresponding to VO_2_Peak > 20, 16–20, 10–16, <10 mL.min^–1.^kg^–1^ which associate with 3 years transplant and mechanical circulatory support-free survival of 97, 93, 83, and 64% respectively (Ritt et al., 2015). Importantly, in heart failure patients with reduced ejection fraction on beta-blockers, VO_2_Peak retains its prognostic significance (O’Neill et al., 2005), and is an important predictor of mortality in heart failure with preserved ejection fraction (Haykowsky et al., 2011; Dhakal et al., 2015). This latter finding is particularly relevant to the renal population given that there is a disproportionate increase in heart failure with preserved ejection compared with reduced ejection fraction in CKD patients (Loutradis et al., 2018). In addition to heart failure, VO_2_Peak has also been shown to be a predictor of survival in chronic lung disease (Nixon et al., 1992; Gitt et al., 2002; Oga et al., 2003; Arena and Sietsema, 2011) and perioperative risk with major surgery (Older et al., 1999; Wilson et al., 2010; Ting et al., 2013).

CPET-derived indices of cardiovascular functional capacity have recently been shown to be robust predictors of cardiovascular morbidity and premature death among patients with advanced CKD, independent of LV measures (Ting et al., 2013, 2014). In a landmark study by Ting et al. (2014) the authors recruited 240 advanced CKD patients who underwent CPET and were followed for ≤5 years. The authors reported that patients with a VT < 40% of predicted peak VO_2_ had a significantly reduced 5 years cumulative survival rate compared with those that had VT ≥ 40% (*P* < 0.001). Significantly, among the patients who had VT < 40%, those that underwent kidney transplantation had a significantly better survival compared with the non-transplanted patients. Among the patients with VT ≥ 40%, survival did not differ significantly between those who were transplanted and those who were not. These results suggest that assessment of VT using CPET can provide a high level of discrimination to risk stratify patients for timely kidney transplantation, and that those with a VT < 40% may benefit from early transplantation. These findings are critical given the growing body of evidence that single surrogate markers from the most established clinicopathologic factors, such as age and LV mass index, have limited prognostic value for cardiovascular outcomes in CKD.

### Physiological Insights

A significant strength of CPET is that the technology provides a wealth of data from a single assessment, including hemodynamics, ventilatory efficiency, stability and O_2_ uptake patterns and mechanical or musculoskeletal parameters. In the general heart failure population, a number of indices have been associated with important measures of cardiovascular physiology such as circulatory power (an index of cardiac systolic function), VE/VCO_2_ (an index of ventilatory efficiency and pulmonary vascular resistance) and mean response time (MRT, an index of right ventricular-pulmonary vascular function during exercise) that can be easily attainable using CPET non-invasively (Malhotra et al., 2016; Mezzani, 2017). CPET assessment, particularly when coupled with hemodynamic, advanced imaging or invasive measures can shed critical insight into the pathophysiology and determinants of cardiovascular alterations, elucidate changes in specific Fick components and provide in-depth insight on the organ limiting gas exchange.

CPET that is coupled with invasive hemodynamic assessment, for example using radial or pulmonary arterial catheters enables highly detailed patient phenotyping. This permits accurate assessment of blood pressure and oxyhemoglobin measurement, as well as simultaneous measurement of VO_2_ and arterial, as well as mixed venous blood gasses during linear ramp exercise. These data will allow Fick cardiac output derivation and evaluation of the component variables of the Fick equation. Patients with identical VO_2_Peak values with a diagnosis of heart failure, may have significantly different levels of impairment in the reserve capacity of each Fick variable (Malhotra et al., 2016). No studies to date have comprehensively assessed CKD patients using invasive CPET, or evaluated how the various Fick variables alter as CKD progresses. By elucidating the mechanisms of cardiovascular dysfunction during progressive CKD, new therapeutic targets for improving cardiovascular outcomes in CKD patients may be identified.

## Conclusion and Future Directions

The significant public health need for better diagnostic and therapeutic approaches to help improve cardiovascular outcomes in CKD requires the implementation of effective, holistic and forward-thinking strategies in our research efforts. Given the multi-systemic nature of CVD development in CKD, an integrative approach at all levels to study mechanisms of disease, to track disease progression or improvement, for risk stratification, and for implementation of appropriate clinical trial endpoints maybe warranted. CPET provides a global test of cardiovascular functional capacity that reflects the entire oxygen transport system and may provide a potential solution for an integrative approach to assessing the cardiovascular system in CKD. The technology provides a wealth of data per single assessment with mechanistic, physiological and prognostic utility. Because a significant advantage of CPET is the ability to couple the technology with other investigative modalities such as invasive CPET and advanced imaging techniques, this can provide comprehensive phenotyping and characterization of multisystem changes that determine overall cardiovascular functional capacity with a wide array of applications. While CPET is a powerful technology, there are also important limitations to the technology that warrants a brief discussion. Firstly, CPET assessment does select for patients who retain some functional ability and therefore, patients who are unable to complete a cycle or treadmill test would be inherently excluded from assessment; Secondly, like all types of exercise testing, patients are required to present to a CPET lab and tests must be conducted by a trained exercise physiologist or clinician. Development of CPET technology has now enabled measurement of ventilatory parameters using handheld devices that can be used in an office-based setting, making the technology increasingly more accessible.

Although CPET is a tool that is now widely available and supported by sound scientific evidence in several clinical fields, only a handful of studies have utilized CPET to study kidney patients. Despite this, CPET has already provided a wealth of hypothesis-generating data and has opened up many other exciting questions in the cardio-renal field. The potential to capitalize on next-generation CPET technology to help move the cardio-renal field forward has provided strong rationale for the urgent need for further CPET-related research in nephrology. Here in the Division of Nephrology at Indiana University School of Medicine, we have developed the first nephrology-based CPET laboratory in the United States dedicated to studying kidney patients. These efforts are in parallel with increasing interest among nephrologists worldwide in exercise impairment and musculoskeletal disorders in CKD patients. CPET technology has the potential to drive actionable strategies in our collective efforts to help reduce the burden of CVD in CKD. Strong efforts to recognize these promising advances and to place them as a primary research agenda in cardio-nephrology are needed.

## Author Contributions

KL wrote the manuscript. GM generated all the figures. AC, GL, and SM provided the intellectual guidance and edited the manuscript. All the authors read and approved the manuscript.

## Conflict of Interest

The authors declare that the research was conducted in the absence of any commercial or financial relationships that could be construed as a potential conflict of interest.
